# Automated Segmentation of the Human Abdominal Vascular System Using a Hybrid Approach Combining Expert System and Supervised Deep Learning

**DOI:** 10.3390/jcm10153347

**Published:** 2021-07-29

**Authors:** Fabien Lareyre, Cédric Adam, Marion Carrier, Juliette Raffort

**Affiliations:** 1 Department of Vascular Surgery, Hospital of Antibes Juan-les-Pins, 06600 Antibes, France; 2 Université Côte d’Azur, Inserm U1065, C3M, 06204 Nice, France; juliette.raffort@hotmail.fr; 3 Laboratory of Applied Mathematics and Computer Science (MICS), CentraleSupélec, Université Paris-Saclay, 91190 Gif-sur-Yvette, France; cedric.adam2@gmail.com (C.A.); marion.carrier&@gmail.com (M.C.); 4 Clinical Chemistry Laboratory, University Hospital of Nice, 06003 Nice, France; 5 AI Institute 3IA Côte d’Azur, Université Côte d’Azur, 06204 Nice, France

**Keywords:** artificial intelligence, machine learning, deep learning, automated segmentation, vascular system

## Abstract

Background: Computed tomography angiography (CTA) is one of the most commonly used imaging technique for the management of vascular diseases. Here, we aimed to develop a hybrid method combining a feature-based expert system with a supervised deep learning (DL) algorithm to enable a fully automatic segmentation of the abdominal vascular tree. Methods: We proposed an algorithm based on the hybridization of a data-driven convolutional neural network and a knowledge-based model dedicated to vascular system segmentation. By using two distinct datasets of CTA from patients to evaluate independence to training dataset, the accuracy of the hybrid method for lumen and thrombus segmentation was evaluated compared to the feature-based expert system alone and to the ground truth provided by a human expert. Results: The hybrid approach demonstrated a better accuracy for lumen segmentation compared to the expert system alone (volume similarity: 0.8128 vs. 0.7912, *p* = 0.0006 and Dice similarity coefficient: 0.8266 vs. 0.7942, *p* < 0.0001). The accuracy for thrombus segmentation was also enhanced using the hybrid approach (volume similarity: 0.9404 vs. 0.9185, *p* = 0.0027 and Dice similarity coefficient: 0.8918 vs. 0.8654, *p* < 0.0001). Conclusions: By enabling a robust and fully automatic segmentation, the method could be used to develop real-time decision support to help in the management of vascular diseases.

## 1. Introduction

Computed tomography angiography (CTA) is one of the most commonly used imaging techniques to evaluate the vascular tree [1,2,3]. Segmentation of the vascular system is of the utmost importance in medical image analysis since the evaluation of the arterial vascularization is useful for the diagnosis of cardiovascular diseases but also represents a critical step to assess the prognosis or plan a surgical intervention in a wide range of diseases including traumatology or oncology [1]. Commercialized software currently available for clinical practice proposes several tools for clinicians to evaluate the anatomy of the vascular system, measure the lengths and the diameters of the vessels. However, none of them are fully automatic and they require human intervention to initiate the localization of the vessels and perform the measurements. Manual segmentation is burdensome, time-consuming and lacks reproducibility due to the intra and inter operator variations. A fully automated method could revolutionize clinical practice by facilitating imaging analysis and by providing quantitative and detailed analysis of the vascular tree.

Several pipelines have been proposed to enable a semi-automatic segmentation of the vascular system thanks to the application of programmed rules searching for known features [4,5,6,7,8]. Such expert systems have the advantage of rationalizing the human process to segment anatomic structures. However, they often require the intervention of a trained expert to initiate the detection and localization of the vascular system, or to provide quantitative measurements of the anatomic characteristics of the vessels.

There is a growing interest in applications of artificial intelligence in cardiovascular diseases [4,9,10,11]. Emerging techniques, including machine learning (ML), and deep learning (DL), especially convolutional neural networks (CNN), have brought new insights into cardiovascular image segmentation [4,9,11,12,13,14,15] and could be applied to develop a fully automatic software. Synthetic data refer to data that are generated by a computer program, instead of being extracted from direct measurement by a human. In medical imaging, they are generally created in order to improve the diversity and increase the effective amount of available data for ML training.

Hybridization corresponds to the process of combining different techniques. Hybrid models are able to combine different techniques (with different rulesets) in the same workflow [16]. Their goal is to overcome the limitations of the individual models, to properly combine a set of predictive techniques in order to improve accuracy of the final prediction. The improvement of accuracy is achieved by combining advantages of individual predictive models and minimizing their disadvantage, at the cost of a higher complexity of the final solution.

In this study, we proposed to evaluate the gain of hybridizing supervised DL algorithm to a feature-based expert system to perform a fully automated segmentation of the abdominal vascular tree in human from CTA-scans. We developed a fully automatic method and compared the results obtained for vascular segmentation with the expert system alone to the hybrid model.

## 2. Materials and Methods

### 2.1. Datasets

The study was conducted according to the guidelines of the Declaration of Helsinki, and approved by the Institutional Review Board of the University Hospital of Nice (protocol n°295, date of approval: 11 October 2018). All methods were carried out in accordance with the French Regulatory Health Authorities and informed consent was obtained from all participants. Two CTA-scan datasets were used to assess the robustness of the methodology and their characteristics are described in Supplemental Table S1.

The first dataset was obtained with CTA-scans from 40 patients with an infrarenal abdominal aortic aneurysm (AAA) and was used in a previous work [17]. CTAs were obtained from patients with fusiform AAA undergoing elective surgery. Syndromic and mycotic aneurysms were excluded. CTA-scan images were obtained from multidetector CT scanners with arterial-phase intravenous injection of contrast liquid. Images were given in Digital Imaging and Communications in Medicine (DICOM) format providing a matrix of size 512 × 512 for each slice with a pixel size of 0.81 +/− 0.13 mm and a slice thickness of 0.90 +/− 0.35 mm. Scans came from 10 institutions and 8 different models of 4 manufacturers (GE Medical Systems, Philips, Toshiba and Hitachi Medical Corporation). The AAA dataset was homogenous, except for six scans that showed arterial systems with an existing stent (including aortic or iliac stent-grafts).

The second dataset included 53 injected CTA-scans at the arterial phase obtained from patients with acute mesenteric ischemia (AMI) and was used for the validation of the algorithms. The etiologies of AMI were classified according to current guidelines into: embolism (*n* = 21), athero-thrombosis (*n* = 18), non-occlusive mesenteric ischemia (*n* = 13), and dissection (*n* = 2) [18]. The AMI dataset exhibited a lower contrast and a larger slice thickness. Images were given in DICOM format providing a matrix of size of 512 × 512 for each slice with a pixel size of 0.84 +/− 0.09 mm and a slice thickness of 2.18 +/− 1.73 mm. Scans came from 5 institutions and 4 different models of 1 manufacturer (GE Medical Systems).

### 2.2. Feature-Based Expert System: Main Programmed Rules

**Figure 1:**
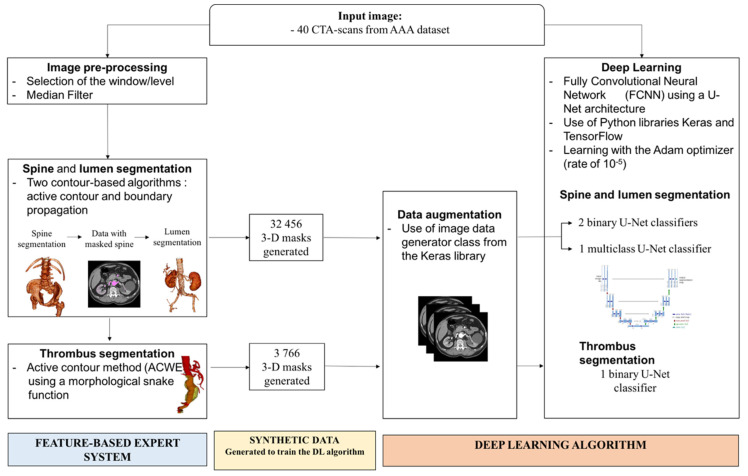
Generation of the deep learning (DL) algorithm.

We used a validated feature-based expert system for segmenting the vascular system on CTA-scans from patients with AAA, as previously described [17]. The pipeline consisted of sequential steps including image pre-processing, spine and lumen segmentations using the active contour and boundary propagation methods, thrombus segmentation with active contour method (Figure 1). This feature-based expert system approach provided the detection of the vascular system including the lumen and the thrombus and discriminates it from adjacent structures such as the spine. The algorithm was fully automated and thus did not need human intervention to segment large datasets of CTA-scans. The computational time varied from 5 to 60 s per CTA-scan.

From the input CTA images, a feature-based expert system pipeline is applied, consisting in 3 sequential steps including image pre-processing, spine and lumen segmentation, and thrombus segmentation. The synthetic data generated by the expert system are used to train the DL algorithm. The DL algorithm uses either a combination of two binary U-Net classifiers, or a multi-class U-Net classifier to segment the lumen and the spine. A binary U-Net classifier is used for thrombus segmentation.

### 2.3. DL Algorithm Architecture

The DL algorithm used was a fully CNN with a U-Net architecture [19]. The Python libraries Keras and TensorFlow were used to construct the model. The U-Net architecture used in this paper is composed of 18 hidden layers in a characteristic symmetric architecture. Two approaches were tested for lumen and spine segmentation: the combination of two binary U-Net classifiers or the use of a multi-class U-Net classifier (Figure 1). For thrombus segmentation, a binary U-Net classifier was used. The total number of trainable parameters was 31,031,691 for the multiclass and 31,031,685 for the binary neural network.

In total, three binary neural networks and one multiclass neural network were generated, segmenting, respectively: lumen, spine, thrombus, lumen, and spine (Figure 1).

### 2.4. Training of the DL Algorithm

The training of the DL algorithm for lumen segmentation was performed on the AAA dataset. The expert system was fed with CTA-scans to generate 3D segmentation masks labelling the regions of interest of the vascular system (*n* = 32,456 images) (Figure 1). The synthetic data corresponding to the CTA-scans and the associated masks were then used to train the DL algorithm.

The AAA dataset was also used for training for thrombus segmentation. The images segmented with the feature-based expert system exhibiting a thrombus were extracted. The images were checked by a human expert who selected images with a high-quality segmentation. Finally, 3766 selected images were used for the training. A sub-dataset of 623 images with thrombus was reserved for testing. Representative images of annotated data generated by the expert system for lumen and thrombus segmentation are available in the Supplemental Figure S1.

The training was achieved using the Adam optimizer with a learning rate of 10^−5^ [20]. The terminating criterion chosen was a stagnation of the Dice for 3 iterations at a 10^−3^ precision. In order to limit the risk of overlearning and increase the robustness, a data augmentation was performed on the learning dataset using the image data generator class from the Keras library.

We compared two approaches for lumen and spine segmentation: two binary U-Net, one dedicated to spine detection and the other to lumen detection, and a multi-class U-Net detecting both spine and lumen classes (Figure 1). For thrombus segmentation, a binary U-Net is used. To evaluate the accuracy of the generated DL algorithms for spine, lumen, and thrombus segmentation, Dice scores over epoch were calculated.

### 2.5. Hybrid Method Combining the Feature-Based Expert System with the DL Algorithm

**Figure 2:**
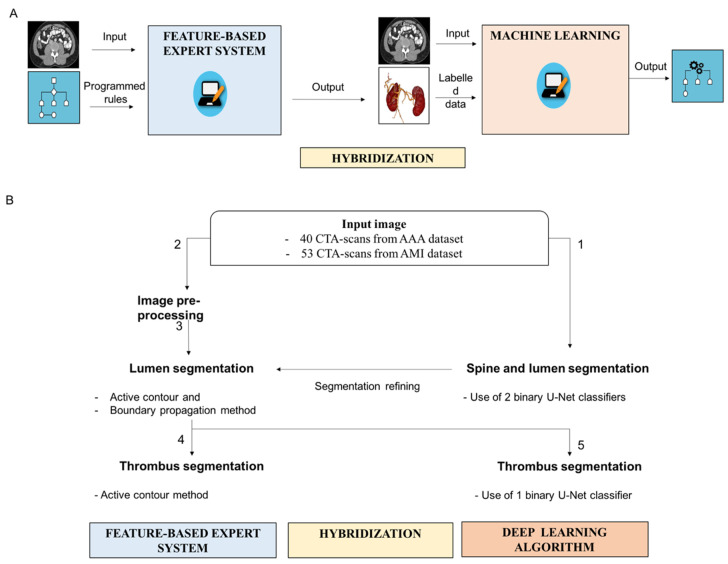
Segmentation of the vascular system using the hybrid approach combining the feature-based expert system with the deep learning (DL) algorithms. (**A**) General principles of expert system and hybrid approach using DL algorithm. In expert systems, input data are processed according to programmed rules to generate output data. In supervised machine learning, the system is fed with input images and labelled data to generate learnt rule. Hybridization consists of combining the two techniques. (**B**) Segmentation of the vascular system using the hybrid approach. The spine and the lumen are segmented using the DL algorithm (1). The lumen segmentation is then refined based on the application of the feature-based expert system following the following steps: image pre-processing (2) and application of active contour and boundary propagation methods (3). The thrombus is segmented using either the active contour method designed in the feature-based expert system (4), or the binary U-Net classifier from the DL algorithm (5).

We developed a hybrid method combining a feature-based expert system with DL algorithm (Figure 2) to segment the vascular system (lumen and thrombus) and enhance its discrimination from the surrounding structures (mainly the spine). General principles of expert system, ML, and hybridization are illustrated in Figure 2A. In expert systems, input data are processed according to programmed rules to generate output data (Figure 2A). In supervised machine learning, the system is fed with input images and labelled data to generate the learnt rule. Hybridization consists of combining the two techniques. In the hybrid model, synthetic data obtained from the expert system are used to train the ML. The ML algorithm can then be combined with the expert system to refine the segmentation.

We employed a DL approach (U-Net architecture) to obtain a first localization of the spine and lumen regions of interest (Figure 2B). The inputs were 2-dimensional images taken along the axial plane. Each input image was an array of size 512 × 512 pixels. The goal of the binary U-Net was to transform each independent input image in a probability map of the same size, with pixel values between 0 and 1. The pixel value represented the probability of belonging to the region of interest. To obtain a binary segmentation mask, we applied a thresholding to the resulting probability map: pixel values lower than 0.5 are set to 0, while the other values are set to 1. The multi-class variation will provide a 3-dimensional map with the third dimension of size equal to the number of classes. As we segmented two classes, representing the lumen and spine, the size of the output map was 512 × 512 × 2.

The spine and the lumen were first segmented using the DL algorithm (Figure 2B). In the CTA-scans, the spine area pixels—detected in the previous sequence—were set to 0. The lumen was segmented a second time on these filtered images using the feature-based expert system to refine the segmentation.

For thrombus segmentation, the feature-based expert system approach used a morphological snake to provide a segmentation mask of the thrombus (Figure 2B). The morphological snake was initialized thanks to the lumen segmented in the previous step. In the hybrid approach using the DL algorithm, the binary U-Net model trained for the detection of the thrombus transformed each independent input image of size 512 × 512 in a probability map with the same size. The pixel value—between 0 and 1—of the map represented the probability of belonging to the thrombus region. To obtain a binary segmentation mask, we applied a thresholding to the resulting probability map: pixel values lower than 0.5 are set to 0, while the other values are set to 1. The diagnosis was cleaned by performing post processing steps. Each connected component that did not intersect the lumen region was removed. An elliptic interpolation was performed to smooth the thrombus area detected in each image along the axial plane.

### 2.6. Methodology for the Evaluation of the Methods

The performances of the feature-based expert system and of the hybrid method using the DL algorithm to segment the vascular system were compared to the ground truth provided by human expert. The manual tracing was performed using a contour drawing method of the image processing software Image J (version 1.52) by one expert vascular surgeon who did not have any access to the results obtained with the automatic methods. The results for lumen segmentation were tested in two datasets: AAA with high resolution and high contrast (*n* = 40) and another independent dataset obtained from patients with AMI with lower resolution and less contrast (*n* = 53). Respectively, 623 and 529 images were manually annotated by medical experts from the AAA and the AMI datasets (Supplemental Table S1). For thrombus segmentation, as the AMI dataset did not exhibit images with thrombus, an independent sub-dataset from patients with AAA was used for testing (*n* = 623 slices) (Supplemental Table S1).

The segmentation results were evaluated using metrics using formulas as previously described [17]: overlap-based metrics (Dice similarity coefficient, Jaccard index, sensitivity, specificity), volume-based metrics (volume similarity) and surface distance-based metrics (Hausdorff distance). Briefly, the Dice similarity coefficient is a measure of the spatial overlap between sets: it ranges from 0, indicating no spatial overlap, to 1, indicating complete overlap [21]. The Jaccard coefficient is a measure of the percentage of overlap between sets: it ranges from 0, indicating no spatial overlap, to 1, indicating complete overlap [21]. The sensitivity measures the portion of positive voxels in the ground truth that are also identified as positive by the segmentation being evaluated [21]. The specificity measures the portion of negative voxels (background) in the ground truth segmentation that are also identified as negative by the segmentation being evaluated [21]. The volume similarity is a measure that considers the volumes of the segments to indicate similarity [21]. The Hausdorff distance is a measure to calculate the distance between two point sets and was calculated according to established formula [22].

Group differences were compared using a paired Student’s *t*-test. A two-tailed *p* value < 0.05 was considered as significant. Statistical analyses were performed using GraphPad Prism software (version 7.00, La Jolla, CA, USA).

## 3. Results

### 3.1. Accuracy and Computational Time of the DL Algorithm Learning

**Figure 3:**
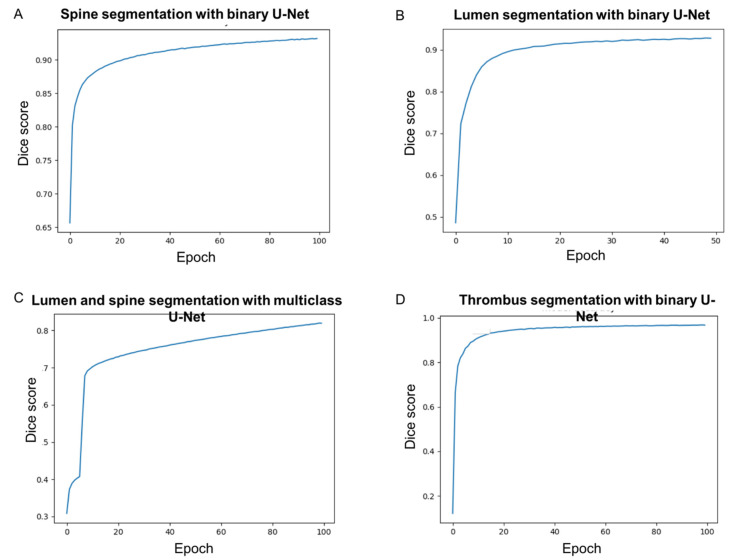
Learning accuracy of DL algorithm for: (**A**) lumen segmentation using the binary U-Net classifier, (**B**) spine segmentation using the binary U-Net classifier, (**C**) lumen and spine segmentation using the multi-class U-Net classifier, and (**D**) thrombus segmentation using the binary U-Net classifier.

Using a binary U-Net classifier, the convergence of the CNN model for spine segmentation was very fast at the beginning of the training and monotonous (Figure 3A). After 100 epochs, the training phase was stopped and the loss based on the Dice coefficient reached 92% (Figure 3A). For lumen segmentation using the binary U-Net classifier, the convergence of the CNN model was also very fast at the beginning of the training and monotonous. After 50 epochs, the training phase was stopped and the loss based on the Dice coefficient reached 93% (Figure 3B). The training lasted 10 hours of elapsed real time using one NVIDIA TITAN RTX graphics card with 4608 CUDA cores at 1770 MHz for each model. The duration of an epoch for the training of the DL network was approximately six minutes.

The convergence of the multiclass CNN model for spine and lumen segmentation was slower than the convergence of the two binary CNN models (Figure 3A–C). After 100 epochs, the training phase was stopped but was converged and the loss based on the Dice coefficient reached 82% (Figure 3C). The evaluation time of the multiclass model was equal to the evaluation time of one binary model. Therefore, the segmentation of the spine and the lumen with the multiclass model was twice as fast as with the binary model. However, the segmented lumen and spine regions proved to be qualitatively less accurate with the multiclass CNN, as shown by lower Dice scores (Figure 3). Therefore, the two binary U-Net classifiers were selected for lumen and spine segmentations for the further design of the hybrid approach. For the thrombus, after 100 epochs, the training phase was stopped and the loss based on the Dice coefficient reached 97% (Figure 3D). The training was performed in two hours using the 2D-U-Net CNN.

### 3.2. Spine Segmentation

**Figure 4:**
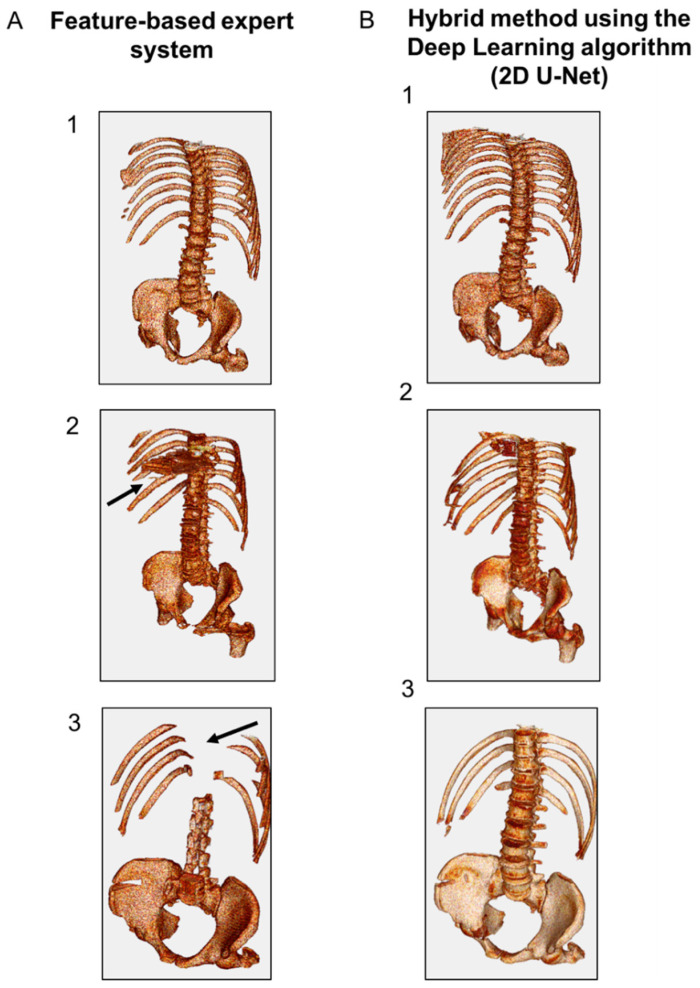
Representative 3D results of spine segmentation using: (**A**) feature-based expert system, and (**B**) hybrid method using the DL algorithm (2D U-Net). (1) Representative images obtained using the AAA data base showing similar performance between the expert system and the hybrid method. (2) Representative images obtained using the AMI database showing false-positive results with the expert system. (3) Representative images obtained using the AMI database showing false-negative results with the expert system.

Qualitative results based on the 3D visualization of the aspect of the segmented spine region were assessed by a human expert for both AAA and AMI datasets (Figure 4). The judgement criterion was the ability of the methods to isolate spine regions close to the vascular system. Using the AAA database, the feature-based expert system and the hybrid method using the DL algorithm (2D U-Net) demonstrated similar performance to detect and discriminate the skeleton in all cases (Figure 4(A-1,B-1)). The AMI database was characterized by lower contrast, larger slice thickness and higher noise. In some cases from this dataset (*n* = 4/53), the expert system provided false-positive results (i.e., visualization of thoracic organs) while the skeleton segmentation was accurate with the hybrid method for all cases (Figure 4(A-2,B-2)). In other cases (*n* = 7/53), the expert system provided false-negative results (i.e., absence of thoracic vertebrae) while the skeleton segmentation was accurate with the hybrid method for all cases (Figure 4(A-3,B-3)).

### 3.3. Lumen Segmentation

**Figure 5:**
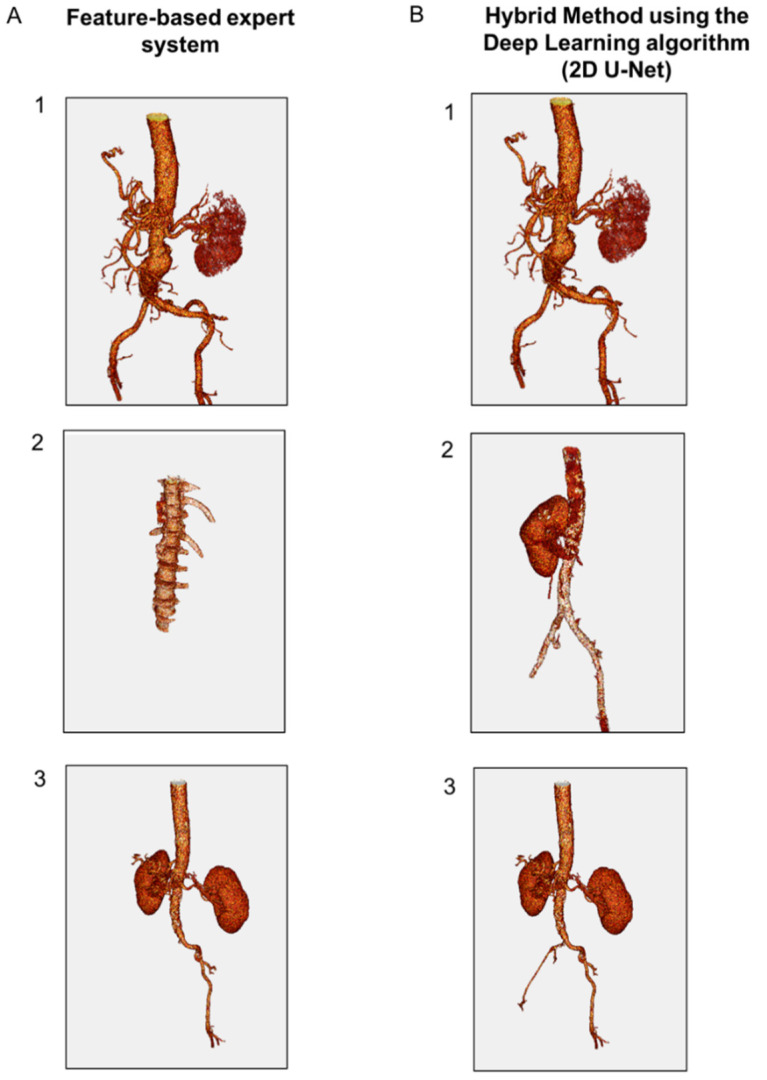
Representative 3D results of lumen segmentation using: (**A**) feature-based expert system, and (**B**) hybrid method using the DL algorithm (2D U-Net). (1) Representative images obtained using the AAA data base. Accurate segmentation by the expert system and the hybrid method (2) and (3) representative images obtained using the AMI database. In low-contrast images, the expert system fails to detect the vascular system while the hybrid approach provides an accurate detection.

Qualitative results based on the 3D visualization of the aspect of the vascular system were evaluated by a human expert (Figure 5). For the AAA dataset, the expert system and the hybrid method using the DL algorithm were able to accurately detect the vascular system (Figure 5(A-1,B-1)). For the AMI dataset, the feature-based expert system failed to identify the vascular system in 5/53 cases (Figure 5(B-1)), while the hybrid method detected it in all cases (Figure 5(B-2,B-3,C-2,C-3)). Hence, the hybrid approach demonstrated a good accuracy to detect the vascular system in both AAA and AMI databases (Figure 5(C-1,C-2,C-3)).

To further compare the performances of the hybrid approach with the feature-based expert system, the results were compared to the ground truth provided by manual segmentation performed by a human expert to calculate the metrics (Table 1). For the AAA dataset, 623 slices were analyzed and both methods demonstrated an excellent correlation of the region measured with the expert ground truth. For lumen segmentation, the mean volume similarity was above 0.90 +/− 0.06 and the Dice similarity coefficient was above 0.92 +/− 0.04 for both methods. For thrombus segmentation, the hybrid method showed better accuracy compared to the expert system for all metrics investigated. The mean volume similarity was 0.8128 +/− 0.2419 for the hybrid method vs. 0.7912 +/− 0.2748 for the expert system (*p* < 0.0006) and the Dice similarity was 0.8226 +/− 0.2354 vs. 0.7943 +/− 0.2774 (*p* < 0.0001). For the AMI dataset, the analysis was performed on 529 slices and demonstrated a better correlation with the ground truth using the hybrid method. The mean volume similarity was, respectively, 0.8128 +/− 0.2419 for the hybrid model and 0.7912 +/− 0.2743 for the expert system (*p* = 0.0006); the mean Dice similarity coefficient 0.8266 +/− 0.2354 vs. 0.7943 +/− 0.2774 (*p* < 0.0001). The computational time between the two methods was similar and ranged from 5 s to 1 min per CTA-scan. For the human manual method, the segmentation time ranged from 25 min to 40 min per scan.

### 3.4. Thrombus Segmentation

**Figure 6:**
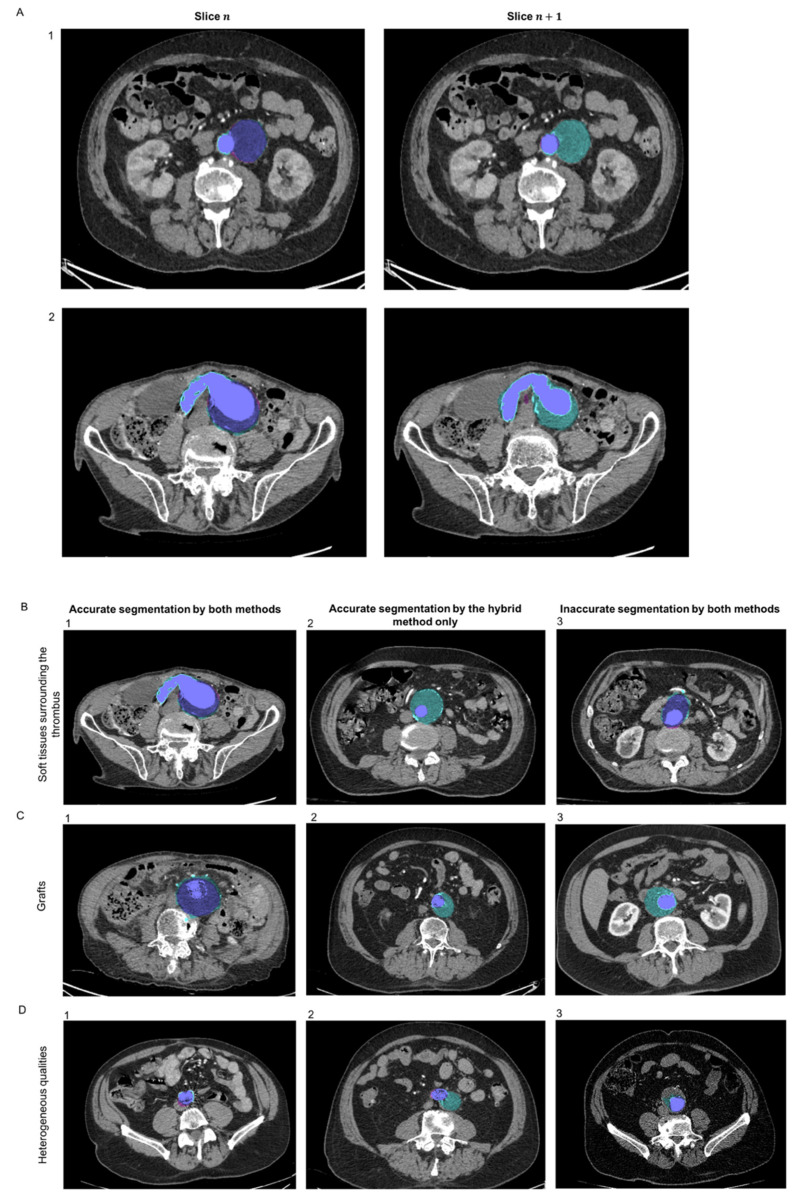
Representative images of thrombus segmentation obtained using the feature-based expert system (magenta pink) and the hybrid method (blue cyan). The merge is colored in blue purple. (**A**) Performance of the methods to detect the thrombus (slice n) and extend the detection over the z axis (slice n + 1) in a case where the section of the aneurysmal has an ellipsoid shape (1) and in a case where the aneurysmal aorta was curved (2). (**B**–**D**) Performances of the method in complex cases—(**B**) cases where the thrombus is surrounded by soft tissues, (**C**) cases with the presence of endografts, and (**D**) cases with poor-quality scans (large slice thickness or low contrast enhancement). (1) Accurate segmentation by both algorithms. (2) Accurate segmentation only with the hybrid method. (3) Inaccurate segmentation by both algorithms.

For thrombus segmentation, as the AMI dataset did not exhibit images with thrombus, an independent sub-dataset from patients with AAA was used for testing (*n* = 623 slices). Representative images of thrombus segmentation in the AAA dataset obtained using the feature-based expert system and the hybrid method using the DL algorithm (2d U-Net) are presented in Figure 6. In the examples provided, both methods exhibited similar performance to segment the thrombus on slice n, while the hybrid method showed a higher robustness on slice n + 1 (Figure 6A). The hybrid method showed higher robustness to detect and extend the thrombus detection over the z axis compared to the expert system. Complex cases were evaluated including situations where the thrombus was surrounded with soft tissues, where an endograft was present or with poor-quality CTA (characterized by large thickness or low contrast enhancement) (Figure 6B–D). Both methods exhibited similar performance to segment the thrombus in some cases (Figure 6(B-1,C-1,D-1)), whereas in others, the expert system failed and the hybrid method showed better robustness (Figure 6(B-2,C-2,D-2)). In rare cases, none of the methods were perfectly accurate to detect the thrombus (Figure 6(B-3,C-3,D-3)). In the case of Figure 6(B-3), both algorithms were close to accuracy to detect the thrombus boundary due to the surrounding tissues and the human expert may also have difficulties to clearly segment it. In the case of Figure 6(C-3), the expert system underestimated the thrombus surface, whereas the hybrid method overestimated it. In the case in Figure 6(D-3), the poor quality of the CTA-scan impeded accurate thrombus segmentation.

Quantitative comparison of the performance between the two methods was performed in the AAA dataset, using manual segmentation performed by a human expert as a ground truth (*n* = 623 slices analyzed) (Table 1). The hybrid method showed a better accuracy than the expert system (ACWE algorithm), as demonstrated by the metrics evaluated (Table 1). The mean volume similarity was, respectively, 0.9404 +/− 0.0775 vs. 0.9185 +/− 0.1575 (*p* = 0.0027) and the mean Dice similarity coefficient was 0.8918 +/− 0.1103 vs. 0.8654 +/− 0.1577 (*p* < 0.0001).

## 4. Discussion

In this study, we developed a hybrid approach combining feature-based expert system with supervised DL algorithm to optimize the accuracy of the automatic segmentation of the abdominal vascular tree.

We trained the supervised DL algorithm on synthetic data generated by the feature-based expert system and tested the results obtained for vascular segmentation with the expert system vs. the hybrid model. The hybrid method demonstrated a similar accuracy compared to the expert system alone in the AAA dataset. The metrics obtained by the two methods for lumen and thrombus segmentation were very close. This could be explained by the fact that the DL algorithm was trained on the AAA dataset labelled by the feature-based expert system. In the AMI dataset, characterized by lower contrast, larger slice thickness and higher noise, the hybrid method demonstrated a better accuracy for vascular segmentation compared to the expert system. A major source of dependency in medical image segmentation is the quality of the image, related to exogenous factors such as machine type, resolution of image, contrast product dose. We chose a holdout validation with a train and test datasets extracted from different pathologies and therefore very different image qualities to assess the generalizability of the method in various pathological settings.

Most of the previously published algorithms dedicated to the segmentation of the vascular system rely on expert systems and Lesage et al. proposed an extensive review of 3D vessel lumen segmentation techniques, with various models, features and extraction schemes [23]. Among them, the region growing approaches and active contours methods are widely used to segment vascular regions [5,24,25]. Connecting morphological operators has advantages compared to traditional numerical solutions such as PDE approaches by being faster, having fewer parameters, and fewer numerical instability issues [26]. We created a feature-based expert system that combined the boundary propagation method with the active contour model to segment the vascular abdominal tree [17]. Siriapisith et al. proposed an automatic detection of the outer wall of AAA using a graph cut based active contour method with a variable neighborhood search that alternates between intensity-based and gradient-based segmentation techniques [27]. The method was tested in 20 CTA obtained from patients with AAA and the mean Dice score was 93.6%. Other investigators proposed a segmentation system that estimates a rough initial surface, and then refines it using a level set segmentation scheme augmented with a global region analyzer and a local feature analyzer [8]. Compared to the ground truth provided by manual tracing on a dataset of 20 CTA, the mean volume overlap was 95.3%. While the above studies demonstrated a good accuracy, the generalizability of the results is limited by the fact that datasets used for testing comprised of a small number of CTA and were all obtained from patients with the same vascular pathology (AAA). In this context, we aimed to improve the expert system by adding DL to allow an accurate detection of the lumen in complex cases and in datasets with very distinct features including patients with another vascular disease (AMI) and CTA with lower contrast.

Several methods using DL techniques have been recently proposed for lumen segmentation in both human and animal models [28,29,30,31,32,33]. Among them, U-Net models have been widely used in medical imaging segmentation [34,35,36,37,38,39]. These techniques are based on deep CNNs that are able to extract deep features. In this study, we trained a CNN with the U-Net architecture to segment the lumen, the spine, and the thrombus. Another study used the U-Net in a very small number of CTA from patients with AAA (*n* = 2) [40]. A cross-validation was used and the Dice scores obtained were over 80% in all the testing subjects. Other investigators aimed to assess the quality of a new automated software for the segmentation of infrarenal AAA [15]. Although the detailed algorithms used are not provided, this software was based on CNN similar to the classical Unet network [15]. Using a homogenous dataset from 100 patients with infrarenal AAA, the comparison between the fully automatic method and manually corrected segmentation for the lumen showed a Dice score of 0.93 +/−0.05. In our study, the Dice score obtained for lumen segmentation from the AAA dataset was in the same range (0.93 +/− 0.04). Nevertheless, the approach and the methodology used in this present study are different. We developed an innovative hybrid model method by taking up the challenge to combine an expert-system method with DL algorithms. In addition, we aimed to test the applicability of the method for lumen segmentation in more complex situations by using other pathological settings and non-homogenous conditions. We thus tested the method in another independent dataset obtained from patients with AMI (characterized by lower contrast, larger slice thickness and higher noise). The results showed the interest of the hybrid model to enhance the performances for vascular segmentation. Finally, other technical perspectives could be added to improve the robustness of the method. In addition to binary U-Net, other DL architectures considering the axial dimension are available and further studies would be of interest to investigate it (i.e., 3D U-Net, 3D-CNN) [41]. Additionally, it would be of interest to train the DL algorithms with more CTAs with a stent graft to increase the robustness of the method in such cases.

The main limitation of a CNN approach resides on the fact that this technique requires a large amount of training data due to the huge number of parameters in the model, usually exceeding several millions of variables to calibrate. To cope with this disadvantage, we created a hybrid model combining the feature-based expert system with the DL algorithm using two binary U-Net classifiers to segment the lumen and the spine. Using synthetic data rather than manual segmentation offer the advantage to be faster, less repetitive, and reduces the potential intra and inter-operator variations. The use of synthetic data generated by the expert system as an input for the DL process creates a continuous improvement loop for the hybrid approach. Other investigators recently proposed a similar approach by training a CNN with images generated with a synthetic shape model in order to segment AAA from CTA-scans dataset [42]. The results showed that the performance of a CNN trained with synthetic data to segment AAAs from new scans was comparable to the one of a network trained with real images. These results are a proof of concept of the interest of a hybrid approach, allowing to generate and train DL algorithms from synthetic data and reducing the burden of obtaining large annotated image databases.

Thrombus segmentation is a challenging task since the borders of thrombus are not well defined. Several expert system methods have been proposed for thrombus segmentation [4,43,44]. Among popular methods, the active contours are commonly used [43,45,46,47,48]. Approaches based on threshold or image gradient may fail since these neighboring objects can distract the method from finding the correct boundary.

Recently, some investigators have proposed a fully automatic detection and segmentation of the abdominal aortic thrombus using a deep CNN [49]. In their study, the networks were trained, validated, and tested in 13 post-operative CTA-scans obtained from patients with AAA. The pipeline achieved a Dice score of more than 82% for post-operative thrombus segmentation. These results highlight the difficulty to reach a high accuracy due to the necessity to acquire enough training data. In our study, the DL algorithm demonstrated a better accuracy with a Dice score reaching 89%. This shows the good performance of the U-Net learnt on synthetic data. This approach offered the advantage to train the dataset with synthetic data selected by a human expert without the need to perform a manual delineation, reducing the time, the burden and the volume of data required for the training.

All in all, vascular segmentation is a very active area and there is an increasing number of start-ups related to AI being created all over the world [50]. Automatic commercialized software is currently being developed and further studies and results can be expected within the next few years, which will probably allow to progressively generalize the use of such applications for daily clinical practice.

## 5. Conclusions

In this study, we used a feature-based expert system previously designed for vascular segmentation [17] to provide large-scale synthetic data generation. We introduced a hybridization by training and confronting DL with the expert system detection. This hybrid method takes advantage of the robustness of the DL approach and provides a more accurate segmentation of the tissues thanks to the precision of the region growing algorithm from the expert system. Such an approach could improve large-scale medical image analysis by improving the quality of the training and reducing the time-consuming task of annotations. By enabling a fast and robust image segmentation, the method could be applied to other vascular regions and be used to develop real-time decision support. Our future research will focus on optimizing the segmentation of the aorta and its main visceral branches to allow an automatic measurement of the anatomic characteristics of the vessels to develop an application for pre-surgical planning. This kind of technology could lead to the development of imaging software that could assist the clinicians to better evaluate the diagnosis and prognosis of patients and help to plan the treatment of vascular diseases.

## Figures and Tables

**Table 1:** Performances of lumen and thrombus segmentation using the feature-based expert or the hybrid method.

**Lumen Segmentation**					
	**AAA Dataset (*n* = 623 Slices)**				
	**Feature-Based Expert System**		**Hybrid Method**		***p* Value**
	**Mean +/− Std**	**Min–Max**	**Mean +/− Std**	**Min–Max**	
Volume similarity	0.9046 +/− 0.0622	0.4475–0.9928	0.9042 +/− 0.0628	0.4475–0.9928	0.1218
Sensitivity	0.9554 +/− 0.0422	0.6751–1.00	0.9550 +/− 0.0424	0.6751–1.00	0.1024
Specificity	0.9995 +/− 0.0003	0.9966–1.0000	0.9995 +/− 0.0003	0.9966–1.0000	0.1075
Jaccard index	0.8681 +/− 0.0691	0.4156–0.9577	0.8674 +/− 0.0696	0.4156–0.9577	0.0245
Dice similarity coefficient	0.9278 +/− 0.0432	0.5871–0.9784	0.9274 +/− 0.0434	0.5871–0.9784	0.0262
Hausdorff distance	6.4491 +/− 10.5375	1.00–83.0241	6.4787 +/− 10.5401	1.00–83.0296	0.0179
	**AMI Dataset (*n* = 529 Slices)**				
	**Feature-Based Expert System**		**Hybrid Method**		***p* Value**
	**Mean +/− Std**	**Min–Max**	**Mean +/− Std**	**Min–Max**	
Volume similarity	0.7912 +/− 0.2743	0.0000–1.0000	0.8128 +/− 0.2419	0.0000–1.0000	0.0006
Sensitivity	0.8334 +/− 0.2765	0.0000–1.0000	0.8854 +/− 0.1823	0.0000–1.0000	<0.0001
Specificity	0.9984 +/− 0.0211	0.5189–1.0000	0.9985 +/− 0.0211	0.5189–1.0000	0.0009
Jaccard index	0.7143 +/− 0.2767	0.0000–0.9076	0.7465 +/− 0.2387	0.0000–0.9584	<0.0001
Dice similarity coefficient	0.7943 +/− 0.2774	0.0000–0.9739	0.8266 +/− 0.2354	0.0000–0.9747	<0.0001
Hausdorff distance	15.4818 +/− 39.8637	0.0000–237.3343	6.0484 +/− 11.4376	0.0000–192.6465	<0.0001
**Thrombus Segmentation**					
	**AAA Dataset (*n* = 623 Slices)**				
	**Feature-Based Expert System**		**Hybrid Method**		***p* Value**
	**Mean +/− Std**	**Min–Max**	**Mean +/− Std**	**Min–Max**	
Volume similarity	0.9185 +/− 0.1575	0.1605–1.0000	0.9404 +/− 0.0775	0.4327–1.0000	0.0027
Sensitivity	0.8594 +/− 0.1664	0.1401–1.0000	0.8665 +/− 0.1457	0.1376–1.0000	<0.0001
Specificity	0.9990 +/− 0.0023	0.9836–1.0000	0.9994 +/− 0.0013	0.9862–1.0000	0.0021
Jaccard index	0.7711 +/− 0.1895	0.1475–0.9648	0.8124 +/− 0.1339	0.1375–0.9595	<0.0001
Dice similarity coefficient	0.8654 +/− 0.1577	0.2447–0.9801	0.8918 +/− 0.1103	0.2337–0.9701	<0.0001
Hausdorff distance	8.9412 +/− 9.3995	2.0000–50.9932	7.5113 +/− 8.8286	1.4131–79.1280	<0.0001

AAA: abdominal aortic aneurysm. AMI: acute mesenteric ischemia.

## Data Availability

All data generated or analyzed during this study are included in this published article.

## Supplementary Materials

The following are available online at https://www.mdpi.com/article/10.3390/jcm10153347/s1, Figure S1: Representative images of annotated CTA generated from the expert system or using manual segmentation by a human expert. (A) Lumen labelling. (B) Thrombus labelling, Table S1: Characteristics of the datasets.

## Abbreviations

AAA	Abdominal Aortic Aneurysm
AMI	Acute Mesenteric Ischemia
CNN	Convolutional Neural Networks
CTA	Computed Tomography Angiography
DL	Deep Learning
DICOM	Digital Imaging and Communications in Medicine
ML	Machine Learning
