# Supplementary material for: Automated Segmentation of the Human Abdominal Vascular System Using a Hybrid Approach Combining Expert System and Supervised Deep Learning
Source: J Clin Med. 2021 Jul 29;10(15):3347. doi: 10.3390/jcm10153347 (PMC8347188; doi:10.3390/jcm10153347)
Supplement: Supplementary file 1 [file jcm-10-03347-s001.zip › jcm-1306174 -supplementary.pdf]

Supplementary Materials

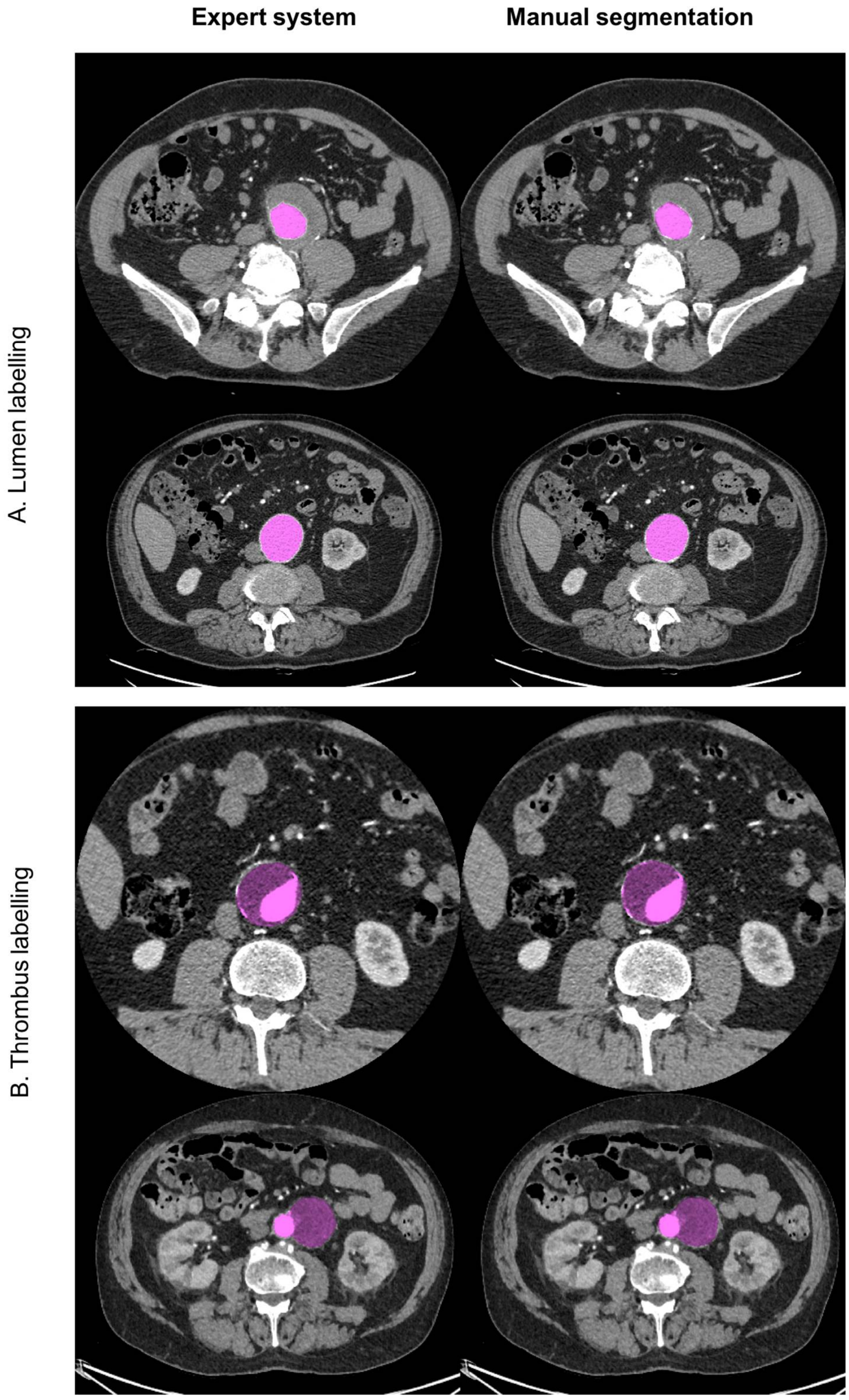

Supplemental Figure S1: Representative images of annotated CTA generated from the expert system or using manual segmentation by a human expert. (A) Lumen labelling. (B) Thrombus labelling.

Supplemental Table S1: Characteristics of the datasets

.	AAA dataset	AMI dataset
Pixel size (mm)	0.81 +/- 0.13	0.84 +/- 0.09
Slice thickness (mm)	0.90 +/- 0.35	2.18 +/- 1.73
Models	10 institutions and 8 different models of 4 manufacturers (GE Medical Systems, Philips, Toshiba and Hitachi Medical Corporation)	5 institutions and 4 different models of 1 manufacturer (GE Medical Systems)
Number of CTA-scans (patients)	40	53
Number of slices per CTA-scans	811 +/- 182	660 +/- 121
Total number of slices	32,456	35,002
Slices manually segmented by a human expert for testing	623	529
Use for lumen and spine segmentation	Training of DL algorithm<br>(32,456 slices numerically segmented by the expert system)<br>Testing of method performances (623 slices manually segmented)	Testing of method performances<br>(529 slices manually segmented)
Use for thrombus segmentation	Training of DL algorithm<br>(3766 slices numerically segmented by the expert system and selected by a human expert)<br>Testing of method performances (623 slices manually segmented)	Not applicable

Values are expressed as mean +/- standard deviation.
